# Modification of 3D Printable Polymer Filaments for Radiation Shielding Applications

**DOI:** 10.3390/polym15071700

**Published:** 2023-03-29

**Authors:** Antonio Jreije, Swaroop Kumar Mutyala, Benas Gabrielis Urbonavičius, Aušrinė Šablinskaitė, Neringa Keršienė, Judita Puišo, Živilė Rutkūnienė, Diana Adlienė

**Affiliations:** Department of Physics, Kaunas University of Technology, Studentu˛ Str. 50, 51368 Kaunas, Lithuania

**Keywords:** 3D printing, fused deposition modeling, polymer composites, radiation shielding, ionizing radiation

## Abstract

There is a growing need to develop lead-free shielding materials that are safe, low weight, durable, environmentally friendly, chemically and mechanically stable and customizable for specific applications. Fused deposition modeling (FDM), an additive manufacturing technique based on the extrusion of a thermoplastic filament into a 3D printed object one layer at a time, could be employed well in applications involving ionizing radiation due to its relatively low cost, design flexibility and high manufacturing precision. This study aimed at developing 3D printing composites that contain Titanium dioxide as a filler agent for shielding in a medical radiation environment. First, the effect of low-dose ionizing radiation (up to 15 Gy) on the mechanical properties of common 3D printing polymers, ABS, ULTRAT, PLA, NYLON, ASA and PETG, was investigated. Since ABS experienced the lowest variation in its ultimate tensile strength (±5%) and Young’s modulus (−5%/+11%), it was chosen as a matrix for a new extruded 3D filament containing TiO_2_ at 1 wt.%, 3 wt.%, and 5 wt.%. With the incorporation of TiO_2_ at different filler contents, the UTS of the ABS composites varied between 24.1 MPa and 28.4 MPa, with the highest value recorded for 3 wt.% TiO_2_. Young’s modulus values were dependent on both the TiO_2_ concentration and on the irradiation dose. In addition, the ABS/TiO_2_ composites with a higher filler content (3 wt.% and 5 wt.%) maintained their attenuation ability even after exposure to a radiation dose of 100 Gy as opposed to pure ABS, which exhibited a ~2.5% reduction in its mass attenuation coefficient after exposure to the same dose of radiation. The pilot investigation performed demonstrated that the newly developed ABS/TiO_2_ composite containing 5 wt.% of filler can be successfully employed to shield electronic devices operating in a radiotherapy room.

## 1. Introduction

Ionizing radiation is often encountered in a wide range of applications, including medicine, energy, industry, agriculture, research and aerospace applications [1,2,3]. As exposure to high-energy ionizing radiation can cause detrimental electronic, chemical and biological effects, the provision of shielding materials that can limit equipment exposure to radiation is of paramount importance [1,2,3,4]. When assessing a material’s performance as a radiation shield, its ability to prevent the penetration of incident radiation through various interaction mechanisms is typically considered. Photons, with a high penetration power, interact with matter through three processes: photoelectric absorption, Compton scattering and pair production. The probability of each interaction is dependent on the photon energy and the composition of the shielding material. For low-energy photon beams interacting with high-atomic-number materials, photoelectric absorption is the predominant process, while pair production becomes predominant for high-energy photons [5]. The interaction of photons with the shielding material is defined by the linear attenuation coefficient (µ), which depends on the energy of the incident radiation and the characteristics of the material. A suitable radiation-shielding material should attenuate radiation with minimal impact on its mechanical, thermal, electrical, chemical and physical properties. Therefore, several factors should be considered when designing radiation-shielding materials: radiation type, radiation energy and intensity and material properties, such as weight, toxicity and environmental impact [6]. Lead and other high-atomic-number (Z) materials are suitable for the attenuation of high-energy radiation [7]. However, it is known that lead has been abandoned from different industries due to its chemical instability and toxic nature, which poses environmental and health risks [8]. In addition, the protective shields manufactured from these materials are heavy and bulky. At the same time, manufacturing is limited in terms of shaping these shields [9,10,11,12,13].

Hence, there is a growing need to develop new shielding materials that are safe, low weight, durable, environmentally friendly, chemically and mechanically stable, have acceptable photon attenuation ability and are customizable for specific applications [14]. Consequently, research is currently being conducted on the development of lead-free radiation shielding using environment friendly materials and technologies. In the last decade, there have been several studies on the use of polymer composites containing fillers of high-atomic-number materials or elements as potential shielding materials. In particular tungsten/epoxy composites [15], Pb_3_O_4_/epoxy composites [16], Gd_2_O_3_/epoxy composites [17], nano concrete composites [5], composites of silicon resin loaded with WO_3_, PbO and Bi_2_O_3_ additives [6] and W- and Ta-containing composites have been considered [18,19]. Tishkevich et al. developed shielding materials to protect crucial components of electronic products and semiconductor units operating in environments that are exposed to high radiation levels. Specifically, they investigated the structure and attenuation coefficients of a WCu composite material in electron and proton radiation settings. The findings demonstrated that WCu composite materials are a highly promising substitute for lead (Pb) in terms of radiation protection [20]. Composite materials typically consist of two components: a polymer matrix and additives. Epoxy has been widely used as the matrix for the composites studied in the field of radiation and nuclear applications because of its good durability and resistance against gamma and neutron radiation [21]. It should be noted that the methodology of producing radiation-shielding materials was usually based on the traditional “casting and molding’’ manufacturing technique in which the composite mix is cast in a silicone mold of the desired shape and allowed to set at room temperature. However, this manufacturing technique has its limitations which include a long setup time, high tooling cost, the need to perform post-processing and the high amount of material waste. These limitations can be addressed through additive manufacturing techniques (AMT), which have the potential to produce a wide range of light-weight, customizable shielding equipment against different environmental and radiation hazards.

Fused deposition modeling (FDM), also referred to as fused filament fabrication (FFF), is one of the most widely utilized additive manufacturing techniques (AMTs). This method involves the extrusion of a thermoplastic filament, layer by layer, to create a 3D printed object based on a computer aided design (CAD) model [22,23]. FDM is characterized by relatively low costs and design flexibility, as well as the high precision and dimensional accuracy of the printed model. One of the key advantages of 3D printing is the ability to create customized and complex objects that would otherwise be difficult or impossible to produce using traditional manufacturing methods [24,25,26,27,28]. With 3D printing, it is possible to produce objects with intricate details that would be too costly or time-consuming to manufacture using traditional techniques. Additionally, 3D printing has the potential to reduce waste and improve sustainability. Traditional manufacturing processes often involve cutting, grinding and shaping materials, which can generate large amounts of waste. In contrast, 3D printing adds material only where it is needed, resulting in less waste and a more efficient use of resources [28]. Most common materials used during FDM are thermoplastic polymers, such as Polylactic acid (PLA), Polyethylene (PE) and Acrylonitrile butadiene styrene (ABS). Due to their superior toughness, high mechanical strength and elevated temperature resistance, these polymers are widely employed in machinery, electronics and aerospace, among other industrial fields [23,24,25,26,27]. FDM-printed models can be recycled back into printable filament, which is in line with current circular-economy trends.

The effect of high-dose ionization radiation on the mechanical and structural properties of polymer systems is well-established [29,30]. The suitability of 3D printing polymers in radiation environments exposed to doses in the kGy and MGy range has been previously investigated [31,32,33]. Wady et al. [34] concluded that the degree of radiation sensitivity varied between 3D polymers, with nylon demonstrating an unchanged ultimate tensile strength (UTS) even at doses of up to 5.3 MGy, while the UTS of all other polymers decreased as function of the radiation dose, reaching a minimum at different exposure doses. Biodegradable PLA was the most sensitive to radiation, showing a 50% decrease in its UTS and Young’s modulus at a relatively lower radiation dose (0.2 MGy) [34]. These changes in the physical properties of organic polymers are caused by chemical reactions driven by radiation-induced radical species. Changes in polymer properties upon irradiation are due to three major mechanisms: crosslinking, chain scission and oxidative degradation [29]. At high doses of radiation, some polymers, including PLA, ABS and PC, undergoes chain scission and oxidative degradation, leading to the radiolytic degradation of the polymers and the formation of low-molecular-weight products, thus contributing to the deterioration of their mechanical properties. Other polymers, such as HDPE and nylon, exhibit crosslinking at a higher dose, leading to an increased stiffness [33,35]. While reviewing the literature, it was evident that previous studies failed to investigate the effect of low doses of ionizing radiation (in the range of few Gy) on the properties of 3D printed polymers. This dose range is typical in medical applications, including radiotherapy in which a linear accelerator (LINAC) is used to deliver a customized beam of high energy X-rays or electrons, targeting a patient’s tumor.

In general, thermoplastics are effectively “transparent” for photons >200 keV and exhibit low mechanical properties [35]. Thus, for a broader application of 3D printable radiation-shielding materials, additives must be introduced into the filament matrix to vary and increase the photon attenuation ability of thermoplastics. Recently, nano- and micro-composite materials, made by dispersing nano- or micro-fillers (e.g., metals, fibers, etc.) in a polymer matrix, have been explored in the field of additive manufacturing [36]. Components manufactured using these composites have been found to possess enhanced mechanical, thermal, and electrical properties in contrast to printed parts made of unreinforced polymer [37]. It should be noted that the characteristics of composite materials are highly influenced by the type of reinforcement used, such as particle, short fiber, or continuous fiber. Additionally, the size, shape, and distribution of the particles’ reinforcement affect the component properties. For instance, in case of nanoparticles, the properties of the composite may be compromised with more than 8–10 wt.% due to the formation of agglomerates. Short-fiber composites exhibit a reduction in properties when loadings exceed 30 wt.%. Previous studies investigated ABS composites reinforced with glass fibers, carbons, graphene, boron nitrate and metal particles for different applications [28,37]. Polymer-based composites also have the potential to be used as radiation-shielding materials since they are lighter than lead-based shielding materials and provide geometric conformability that is similar to other structural materials (i.e., metals and alloys) commonly used in various industries [37].

The aim of this study was to develop new polymer composites for 3D printing that contain Titanium dioxide as a filler agent, to investigate their shielding ability and mechanical properties and to conduct a pilot study on the applicability of a 3D printable, polymer-based radiation-shielding construction for the protection of electronic devices in a medical radiation environment.

## 2. Materials and Methods

### 2.1. Testing of Commercially Available Thermoplastic Materials

To test the feasibility of using 3D printed shielding structures in the radiation field, a series of experiments was performed. Firstly, six sets (7.5 cm long strips) of commercially available 3D printing filaments (ABS, ULTRAT, PLA, NYLON, ASA and PETG), all purchased from Zortrax (Olsztyn, Poland), were prepared for the investigation of the impact of radiation on their mechanical properties (Table 1) in order to determine the most suitable candidate for the formation of radiation-shielding constructions. For this purpose, the filament strips were irradiated at different doses from 2 to 15 Gy in a linear accelerator, Clinac DMX (Varian, Palo Alto, CA, USA), using a 6 MeV X-ray beam.

**Figure 1:**
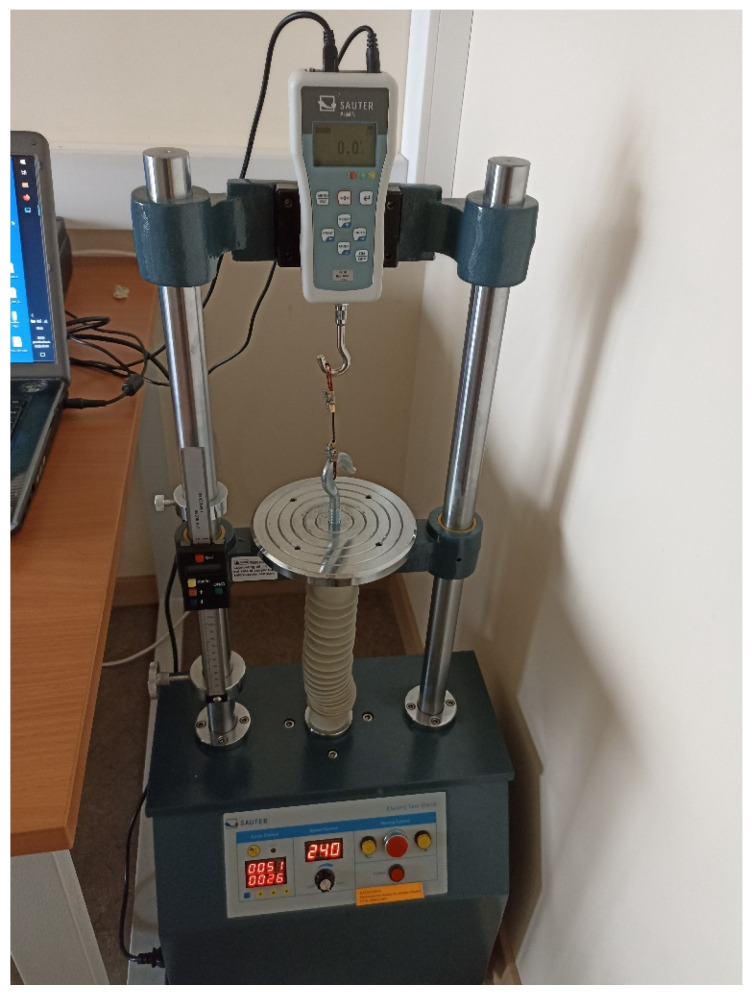
Test bench for mechanical testing; Sauter TVM 5000N230.

To assess the possible radiation-induced changes in the mechanical properties of the irradiated filaments, their ultimate tensile strength (UTS) and Young’s modulus (YM) were considered the most important parameters for the evaluation. The tensile deformation of samples was performed using a test bench Sauter TVM 5000N230 (Figure 1).

Each filament was stretched at a constant velocity of 4.2 mm/s until breaking. The force (F) applied to the filament and the elongation (∆L) of the filament due to deformation were measured throughout the test. For each tensile test, three samples were used (i.e., 3 non-irradiated controls and 3 samples irradiated with appropriate doses) to achieve a statistically representative sample.

### 2.2. Extrusion FDM Filaments

ABS polymer was selected for the further development and investigation of new composite materials for 3D printed radiation-shielding constructions. The selection of ABS was based on a literature review and on the results of the mechanical tests performed on the irradiated samples, which are provided in the “Results” section (Section 3.1). ABS is the most common 3D filament material on the market. At the same time, the properties relating to its extrusion into 3D filaments from granules is well-known. TiO_2_ powder (~50 μm, 98%, Sigma Aldrich, Taufkirchen, Germany) was selected as an additive due to its relatively high radiation attenuation properties when in pure form. Additionally, TiO_2_ is readily available in a powered form, which is practical when mixing with the ABS.

**Figure 2:**
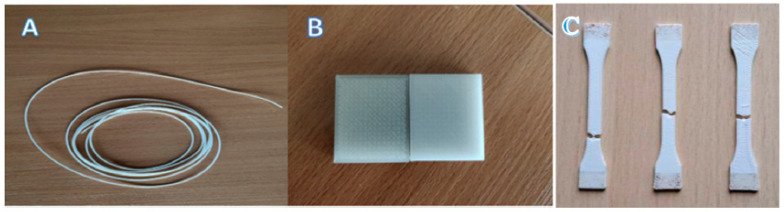
Experimental samples: (**A**) extruded ABS filament; (**B**) 3D printed ABS samples with different concentrations of TiO_2_ fillers for attenuation experiments; (**C**) 3D printed ABS samples (ISO527-2 specimen 1A) containing different concentrations of TiO_2_ after tensile stress and strain tests.

For the production of the 3D printable filament, ABS granules were dry-mixed with different amounts of TiO_2_ additive (1%, 3% and 5%). Using a screw extruder (Precision 350, 3Devo Filament Maker, Eindhoven, The Netherlands) (Figure 2A), filaments with a diameter of 1.75 mm were extruded in 50 g batches. A standard 3Devo material preset for ABS was selected for extrusion, taking into account that the extrusion device had four heating zones: preheating (240 °C), melting (230 °C), shear (220 °C) and extrusion (215 °C). The transition temperature of ABS from solid to liquid was 215 °C. Spooling was only initiated after the extruded filament reached a stable diameter. 

### 2.3. Preparation of 3D Printed Samples

Two series of samples from the produced filaments were prepared for further experimentation: the evaluation of the mechanical and radiation attenuation properties of the samples.

To determine changes in the radiation attenuation properties of the produced polymer composites (bulk material), experimental samples at a size of 50 × 50 × 5 mm were printed from each filament type using a 3D printer (Zortrax M300, Olsztyn, Poland) (Figure 2B). It is important to note that during the FDM printing volume, infill ratio plays an important role in terms of attenuation properties. Any infill ratio lower than 100% will produce cavities in the printed volume which will drastically decrease the attenuation coefficient value. To avoid this effect, only samples printed with a ratio of 100% were used in further experiments. The printing temperature was set to 260–290 °C, depending on the amount of additive (the printing temperature increased with an increase in the concentration of the additive). Additionally, the bed temperature was set to 80 °C in order to avoid the warping that might occur with ABS.

In order to determine the tensile properties of the new composite materials, dog-bone-shaped specimens were 3D printed according to ISO527-2 standard (type 1A specimens), using composite filaments containing different concentrations of TiO_2_ (Figure 2C).

### 2.4. Measurement of Radiation Attenuation Properties of Polymer Composite

Gamma irradiation of the samples was carried out using a Cobalt-60 (1.173 and 1.332 MeV) source. The plates were positioned between the ^60^Co source and the radiation detector, with a source-to-detector distance of 5 cm. The reading was performed using a Gamma Scout Geiger counter (Gamm-Scout GmbH & Co. KG, Cologne, Germany). The linear attenuation coefficient was determined using the Beer–Lambert law, according to the following equation:

I = I_0_e^−µx^ (1)

where I and I_0_ are the primary incident and transmitted gamma intensity, μ is the linear attenuation coefficient of the material at a specific energy and x is the thickness of the material [38]. The mass attenuation coefficient (μ/ρ) was obtained by dividing the linear attenuation coefficient μ by the density ρ of the material.

### 2.5. Performance of Mechanical Testing

**Figure 3:**
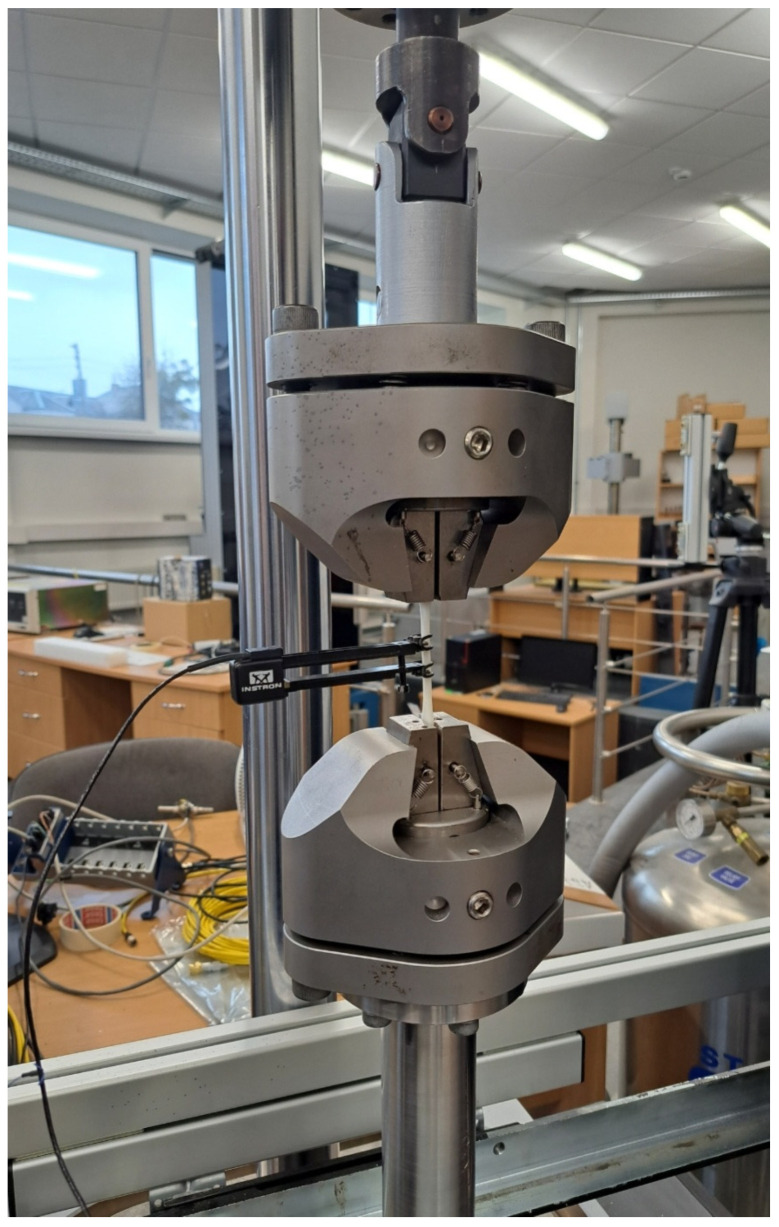
Mechanical testing machine: ElectroPuls^®^ E10000 Linear-Torsion.

Tensile tests of the 3D printed samples were performed using a dynamic and static mechanical testing machine, the ElectroPuls^®^ Linear-Torsion (Instron, Norwood, MA, USA) (Figure 3), which includes Instron^®^ advanced digital control electronics, a bi-axial Dynacell™ load cell, console software, electrically operated crosshead lifts, a T-slot table for flexible test setups and very advanced, hassle-free tuning based on specimen stiffness. The system is designed to study the linear (10 kN, ±30 mm) and rotational motion (100 Nm, ±135° or ±16 revolutions). Sample specimens were fixed in self-tightening clamps and extended along the sample’s major longitudinal axis at a constant test speed of 1.00000 mm/min until the specimen fractured. The gauge length was L_0 = 25 mm. The load sustained by the specimen and the elongation were measured during these tests.

### 2.6. Investigation of Material Applicability for the Radiation Shielding of Electronic Devices

The applicability of the developed composite filament as a shielding material was tested out by evaluating its efficacy in protecting electronic equipment against ionizing radiation. A shielding case was printed for a compact digital camera from the newly developed ABS/TiO_2_ composite filaments (at a TiO_2_ filler concentration of 5 wt.%). The modeling and design software SOLIDWORKS (Dassault Systeme, Vélizy-Villacoublay, France) was used for creation of a virtual case model, which was converted into a stereolithography file format (.stl) suitable for 3D printing. The protective case was designed to fit perfectly and to cover the whole camera body except for the lens. The 3D printing of the case was performed using following printing parameters: nozzle temperature—270 °C; build plate temperature—80 °C; infill density—100%; layer thickness—0.29 mm.

**Figure 4:**
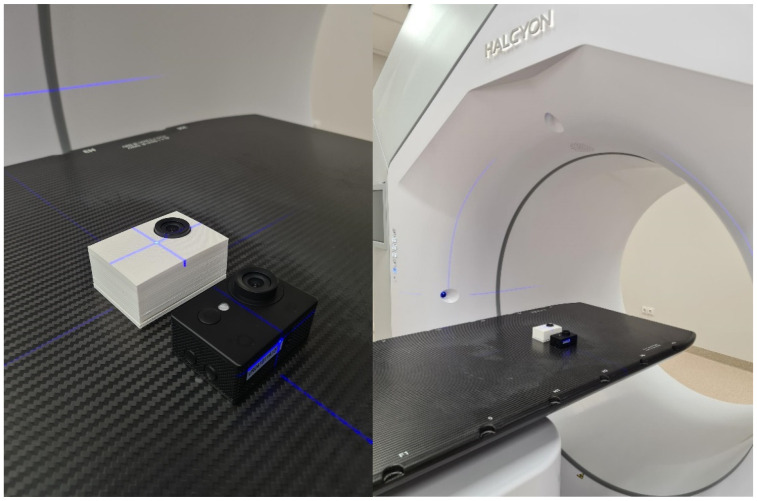
Irradiation of two cameras with and without protective casing, using a linear accelerator.

The investigation involved the irradiation of two identical digital cameras (the same as those used in radiotherapy treatment rooms) with 100 Gy dose at a dose rate of 5 Gy/min using a Halcyon Linear accelerator (Varian, USA) (Figure 4). One camera was inserted into the 3D printed radiation-shielding case and the other was left without any protection. Both cameras were irradiated simultaneously and were turned on during the experiment to better reflect the real-world scenario.

A dead pixel count in the images captured was chosen as a quantitative measure of radiation protection provided by the shielding. For this reason, a set of dark images were captured to determine the % of dead pixels in the imaging data after the irradiation of both digital cameras. Image processing was performed with the open-source image processing software ImageJ (Fiji), using a threshold and particle count. In order to avoid bias, baseline camera sensor functionality was assessed before the experiment.

## 3. Results and Discussion

### 3.1. Radiation-Induced Degradation of Mechanical Properties of Commercially Available 3D Printing Filaments

In order to evaluate the tendencies of the radiation-induced changes in the mechanical properties of commercial filaments, the ultimate tensile strength (UTS) at a corresponding elongation and the average Young’s modulus (YM) for each non-irradiated material were estimated in a first step (Table 2).

**Figure 5:**
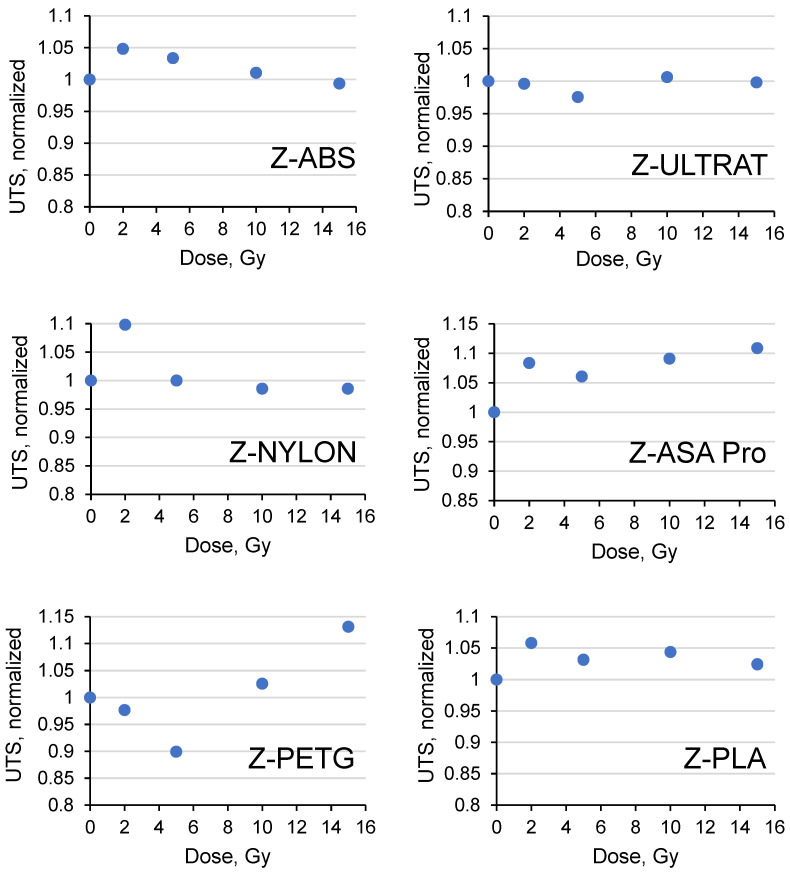
Variations in ultimate tensile strain (UTS) of the irradiated 3D printed filaments.

**Figure 6:**
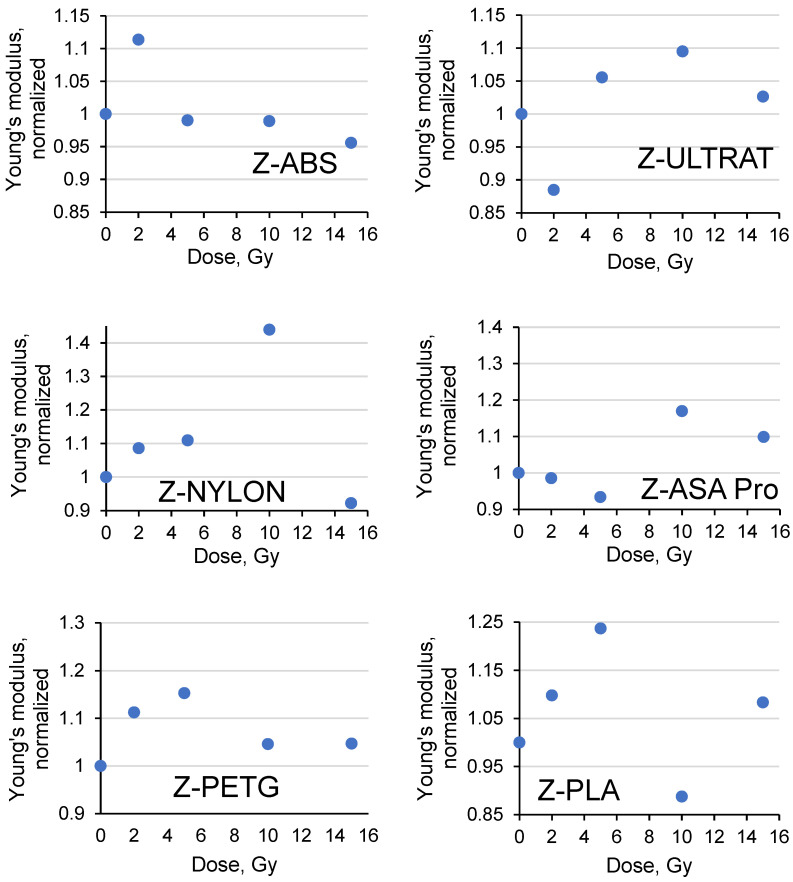
Variations of Young’s modulus of irradiated 3D printed filaments.

In a second step, similar UTS and YM measurements were performed for the filaments irradiated with 6 MeV energy photons in doses of up to 15 Gy. The obtained results were normalized to the corresponding values of the non-irradiated samples and are provided in Figure 5 and Figure 6.

It should be noted that in this study, the tensile testing of the commercial 3D printed filaments was performed directly on the filaments, while other authors investigated the mechanical properties of the 3D printed specimen [39,40,41,42,43]. This methodology was implemented with the aim to eliminate any dependency of mechanical performance on the 3D printing parameters (i.e., layer thickness, infill patterns and part geometry, number of contours/shells, strength of welding zones within a layer, etc.).

The analyses performed revealed that the investigated commercial filaments demonstrated good performance in the dose range of up to 15 Gy, and the irradiation had only a very modest impact on their mechanical properties. It was found (Figure 5) that the normalized values of the ultimate tensile stress (UTS) for the ABS, ULTRAT and PLA samples varied within ±5%, and the UTS values for nylon and ASA Pro varied within ± 10% intervals. There were no specific tendencies related to the increased dose observed except for the PETG filaments. The UTS values of PETG increased slightly with the increasing irradiation dose and varied significantly within the range between −10% and +15%.

The Young’s modulus values calculated (Figure 6) varied in broader intervals when compared to the UTS values: the lowest YM variations were observed for the irradiated ABS (−5%/+11%), ULTRAT (−11%/+9%), PETG (0/+15%), nylon (−9%/+14%), ASA pro (−9%/+17%) and PLA −9%/+23%. It should be noted that the Young’s modulus values of the investigated samples were calculated from the data obtained during the mechanical tests; thus, additional errors related to possible measurement/evaluation uncertainties should be considered. It was also suggested that the small variations in the UTS and YM values were caused by specific, radiation-induced crosslinking and scission processes in polymer filaments that are influenced by the slightly different chemical compositions and molecular structures of the irradiated species.

Based on the results of the performed investigation, the ABS sample indicated the lowest impact of radiation on its properties. Therefore, this material was chosen for the polymer composite extrusion experiments and further investigation.

### 3.2. Characterization of the In-House-Produced ABS Filament with Additives

The key to the success of FDM depends upon the proper selection of printing parameters (e.g., layer thickness, road width, fill density, deposition speed, nozzle diameter, etc.) and on the filament’s quality. Moreover, FDM requires filaments of a uniform and round diameter since variations in the filament cross-section and diameter will result in poor printing quality or even failures [44,45]. This geometrical constraint was used to guide the maximum weight percentage of TiO_2_ filler that can be added while producing a filament of good quality. In this investigation, 1 wt.%, 3 wt.%, and 5 wt.% infill concentrations were used for the successful extrusion of new filaments. Since the filler concentration may influence the radiation attenuation properties of the material, multiple unsuccessful printing attempts have been made in trying to extrude filaments that contain a higher concentration of fillers. This failure in printing is most likely attributed to the filament size variation and the overfilling of filaments with powder, which resulted in the printer nozzle clogging. Taking into account that the 3D filament extrusion method applied in this study was based on dry-mixing the ABS polymer with TiO_2_ powder before feeding the mixture into the extruder feed hopper, the total amount of additive that could be added to the polymer matrix and result in the sub-optimal uniformity of the additive distribution was limited. A similar extrusion technique was used by Vidakis et al. [39], who investigated the mechanical properties of ABS and TiO_2_/ATO nanocomposites. This author succeeded in fabricating an ABS composite with higher concentrations of TiO_2_ (up to 10% as opposed to the 5% used in this study) due to the following factors: ABS powders were used instead of granules, which have a larger surface area and thus a better interaction with the filler powder, and the TiO_2_ powder had a smaller particle size (in the range of 25–50 nm) [39].

The filler material investigated in this study (TiO_2_) was chosen based on its wide availability and low cost. The additives had a particle size of ~50 μm. Moreover, the concentration of the added TiO_2_ influenced the 3D printing temperature. This was taken into account, and the temperature was gradually increased from 260 °C to 280 °C when working with pure ABS and the polymer composite filler (Table 3). It was assumed that the increased filler concentration (>3%) may have had an effect on the glass transition temperature (T_g_) of the ABS due to the formation of filler agglomerates inside the polymeric matrix, which resulted in the considerable modification of the ABS sub-molecular structure. It should be noted that in the previous studies, the T_g_ of the ABS/TiO_2_ nanocomposites (~95 °C) was found to be lower than that of pure ABS samples (102 °C) [39,40,41]. The predominant cause for the decreased T_g_ in the ABS containing TiO_2_ could be the repulsive interaction between the nanoparticles and the surrounding polymer, which leads to an increase in free volume and chain mobility near the nanoparticles [39,40,41]. However, at a higher percentage of added TiO_2_, aggregation between particles increases, leading to fewer interaction between the particles and the polymer matrix and consequently increasing the T_g_.

### 3.3. Investigation of Radiation Attenuation Properties of Newly Developed Polymer Composites

**Figure 7:**
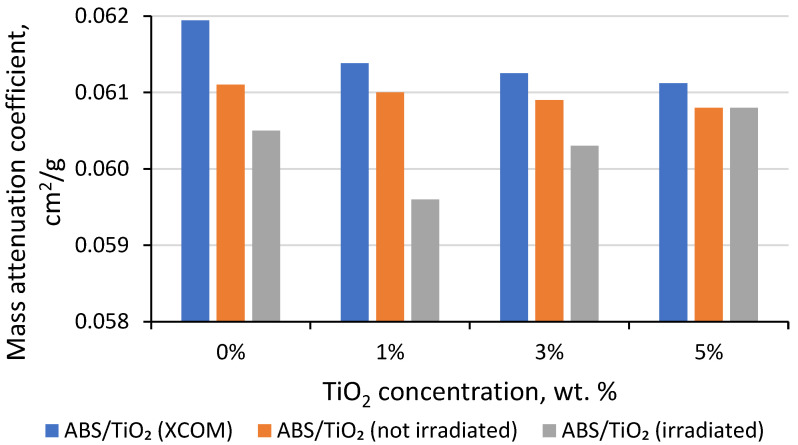
Mass attenuation of ABS samples with different concentrations of TiO_2_ fillers.

It is known [30,31,32,33,34,35] that the prolonged exposure of objects to high doses of radiation may cause the radiation-induced degradation of the irradiated material’s properties. This is also valid for 3D printed materials used to shield equipment used in radiation treatment rooms (video cameras in our case). Three-dimensionally printed materials should be radiation-resistant, and the alteration of their radiation attenuation properties due to exposure to high doses of radiation should be minimal. The results of gamma ray (1.25 MeV) attenuation measurements in 3D printed pure ABS and in ABS containing different concentrations of TiO_2_ are presented in Figure 7, along with the results of simulations performed using the NIST XCOM database [46]. It should be noted that the radiation attenuation in materials was measured twice: in the prepared 3D printed samples and in the same samples after their irradiation with a 100 Gy dose.

It was found that the attenuation ability of the printed materials was lower when compared to the theoretical evaluations obtained by exploring the NIST XCOM database [46]. However, differences between the mass attenuation coefficients of not-irradiated and irradiated samples were small, resulting in deviations of <2.5% between values. A possible explanation for the small reduction in the attenuation ability of the printed samples could be related to the specificity of the polymer composites’ extrusion technique, which resulted in some inhomogeneities of filler distribution in the composite matrix. It is also possible that the voids are created due to the vastly different melting points of the mixed materials. The decrease in the radiation attenuation with an increase in the filler’s concentration of up to 5 wt.% may be attributed to a possible agglomeration of filler powder particles. However, it can be seen that the samples irradiated with a high dose showed a modest increase in their attenuation ability with the increase in the filler concentration, which might be explained by the possible radiation-induced additional polymerization of the thermo-polymeric 3D printing materials. A comparison of the attenuation properties of the 3D printed materials and Pb has shown that the attenuation properties of a >3 mm thick ABS-TiO_2_ (5%) composite material plate would be as sufficient to protect equipment against ionizing radiation as any other material providing an equivalency to 0.25 mm Pb.

### 3.4. Radiation Impact on Mechanical Properties of New Composites

**Figure 8:**
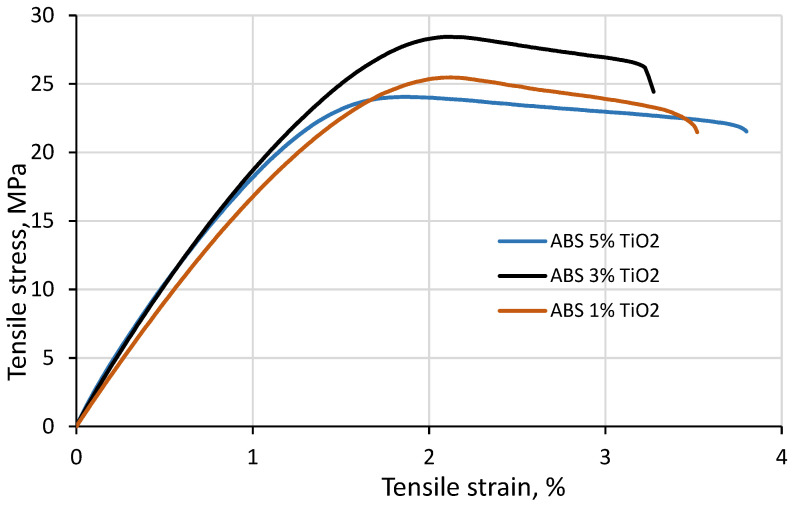
Tensile stress and strain diagram of non-irradiated 3D printed composite samples.

It is expected that the properties of pure material filaments will be different from filaments containing TiO_2_ additives. The same is valid for 3D printed objects compared to filaments, even if printing is realized with 100% filling. These deviations are related to the inhomogeneities of the extruded filaments that may occur. Due to the reasons mentioned above, the mechanical properties (elongation and deformation) of the 3D printed composite specimens printed at an infill ratio of 100% were investigated. The investigation performed covered non-irradiated and 3D printed composite samples irradiated with up to 70 Gy. The mechanical properties of the investigated samples are summarized in Table 4, and an example of the obtained and analyzed sample-related tensile deformation curves is provided in Figure 8.

It was found that the UTS values for the 3D printed ABS/TiO_2_ composites were generally smaller when compared to the initial commercial filaments (25.1 MPa vs. 40.1 MPa, respectively). This could be explained by the fact that the extruded polymer composite filaments were inhomogeneous not only in terms of filler distribution within the polymer matrix but also in terms of the filament’s shape due to the possible agglomeration of the filler particles, leading to the formation of small, air-filled voids and pores in the 3D printed samples.

The tensile strength of the ABS/TiO_2_ composites at 1% wt. did not vary from pure ABS (25.07 vs. 25.48 MPa at 0% and 1%, respectively). AT 3% wt., the tensile strength increased to 28.44 MPa, and at higher filler concentrations, a decrease in the composite’s tensile strength was observed (24.06 MPa at 5 wt.%). Changes in the composite’s tensile stress can be explained by the interaction of the filler particles with the polymer matrix. The effective size of the filler affects the mechanical properties of the final composite. As the filler concentration increases, the particles clump together, leading to a larger effective particle size and a lower volumetric particle surface area. Consequently, the number of particle–polymer interactions decrease, and the polymer strength increases due to the increased polymer flexibility near the particle’s aggregates or due to the intercalations of polymer chains. Beyond a certain filler concentration, the polymer chains become immobilized, with a high concentration of stress upon the points of aggregation. This induces fracture points and therefore diminishes the mechanical properties of the composite.

There were not very significant variations in the UTS values introduced by changing the filler concentration or irradiating the samples with 6 MeV photons up to 70 Gy. However, some results fit well with the results achieved by other authors. For example, Vidakis et al. [39] reported a 7% increase in the tensile strength of the ABS/TiO_2_ composite at the 2.5 wt.% TiO_2_ filler concentration when compared to pure ABS, which was followed by a decrease in tensile strength at 5 wt.% and 10 wt.% TiO_2_ [39]. The results of this research indicated a 13% increase in tensile strength for the not-irradiated ABS/TiO_2_ composite at 3 wt.% of filler and 4% decrease at 5 wt.% of filler when compared to pure ABS.

**Figure 9:**
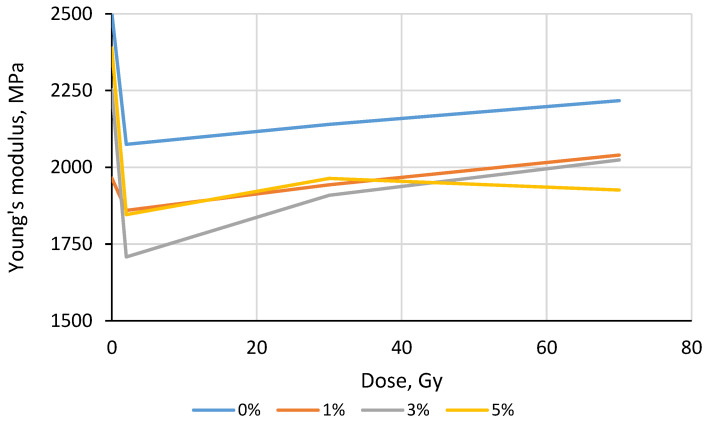
Variations in Young’s modulus of irradiated ABS samples with different concentrations of TiO_2_ filler.

The calculated Young’s modulus values were slightly dependent on TiO_2_ concentration and on the irradiation dose, as can be seen in Figure 9.

The highest YM values were obtained for the pure, non-irradiated ABS samples and were slightly decreased when the concentration of filler increased. The initial 2 Gy irradiation caused a reduction in the YM values of all 3D printed samples. The further increase in the irradiation dose led to steadily increasing Young’s modulus with the exception of the ABS composite containing 5 wt.% of TiO_2_ filler, which indicated variations in the YM values of an approximately average value.

The research performed demonstrated small variations in the UTS values of different samples and a slight tendency of the Young’s modulus to grow with an increasing irradiation dose for all samples except for the ABS/5%TiO_2_ samples. Taking into account that the last sample indicated the best radiation attenuation ability and that its Young’s modulus value was altered at an approximately average value due to irradiation of up to 70 Gy, this sample was to design a shielding element for a video camera operating in a radiotherapy room.

### 3.5. Three-Dimensionally Printable Radiation Shielding

**Figure 10:**
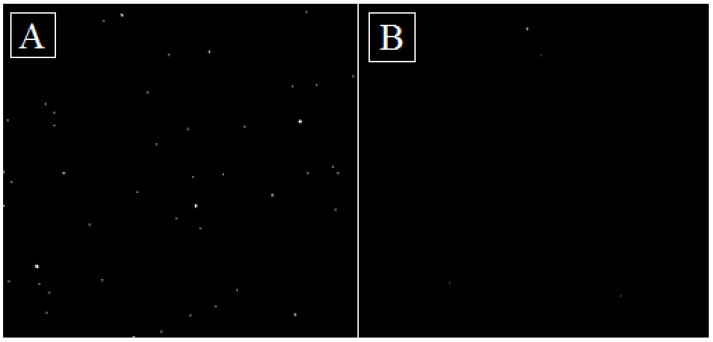
Threshold image captured with (**A**) unprotected camera and (**B**) protected camera.

The effectiveness of the developed 3D filament at shielding electronic equipment was investigated. Electronic devices are available in different environments that use ionizing radiation, including the medicine, energy, industry, agriculture, research and aerospace industries. A case scenario of a camera operating in a radiotherapy room containing a linear accelerator was simulated. In such an environment, these cameras are usually exposed to around 100 Gy per year of continuous operation. The camera-shielding case was printed from extruded ABS filaments containing 5 wt.% of the TiO_2_ composite. Irradiation was performed in a linear accelerator (Figure 4), and the radiation (100 Gy)-induced damage to a digital camera placed in a 3D printed shield was compared to the damage sustained by a non-protected camera. The most critical hardware component in a digital camera is the sensor, which consists of an array of millions of photosites that collect photons and convert them into an electrical signal to compose an image. Damage to any of these photosites results in a dead pixel in the captured image. Images captured by the protected and non-protected cameras are shown in Figure 10. It should be noted that the baseline camera sensor functionality showed no dead pixels in either digital camera. The results show that the protected camera has a visibly lower dead pixel count. By counting the total particles in the image, it was found that the unprotected camera produced an image with 23% more dead pixels. This shows that it is possible to use the ABS/TiO_2_ printable composite to design simple, low-cost 3D printed radiation-shielding equipment for some electronic devices used in radiation treatment rooms, thus achieving measurable improvement in radiation shielding.

## 4. Conclusions

The range of commercial 3D filaments included in this study behaved differently in terms of their UTS and YT after exposure to ionizing radiation, even though the changes were not significant at the low-dose interval usually encountered in a medical application (<15 Gy). The incorporation of TiO_2_ additives into the ABS polymer matrix at a 3 wt.% improved the UTS of the ABS composite by 13%, while a 4% decrease was observed at 5% of filler concentration. Moreover, the ABS/TiO_2_ composites with a higher filler content maintained their attenuation ability even after exposure to a radiation dose of 100 Gy, while the attenuation property of pure ABS was reduced by ~2.5% after exposure to the same dose. Since the ABS/TiO_2_ composite at 5 wt.% of filler showed favorable radiation attenuation ability and Young’s modulus values that were altered around average, even after irradiation with up to 70 Gy, this composite was used to manufacture shielding for a digital camera located in a linear accelerator treatment room. The integration of 3D printing into the design and manufacturing of a shielding component using the newly developed composites was shown to be effective in protecting electronic devices against radiation-induced damage. Through this technique, custom-made, lighter shielding devices can be produced at a lower cost and with less material waste. Overall, this approach of the in-house-fabrication of a 3D composite is very promising, although changes to the extrusion method should be made in order to improve the homogeneity of the additive’s distribution in the polymer matrix.

## Figures and Tables

**Table 1:** Samples of commercially available 3D printing filaments.

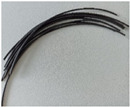	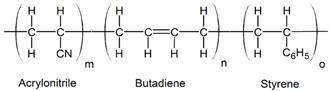
Z-ABS (*acrylonitrile-butadiene-styrene copolimer)*	
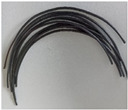	ABS plastic blend
Z-ULTRAT (*acrylonitrile-butadiene-styrene copolimer based)*	
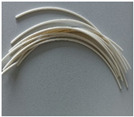	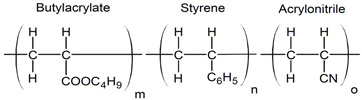
Z-ASA Pro (*acrylonitrile-styrene-acrylate)*	
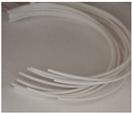	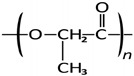
Z-PLA *(polylactic acid)*	
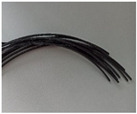	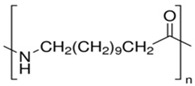
Z-NYLON	
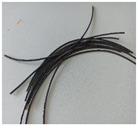	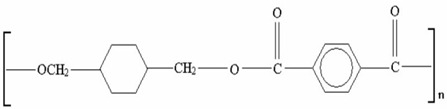
Z-PETG *(Polyethylene terephthalate glycol)*	

**Table 2:** Measured mechanical properties of commercial 3D printing filaments.

Filament Material	Density, g/cm^3^	Tensile Strength, MPa	Young’s Modulus, MPa
Z-ABS	1.04 ± 0.02	40 ± 10	1869 ± 35
Z-ULTRAT	1.08 ± 0.12	42 ± 4	1950 ± 29
Z-NYLON	1.03 ± 0.11	40 ± 5	1870 ± 112
Z-ASA Pro	1.07 ± 0.92	55 ± 5	2020 ± 37
Z-PETG	1.23 ± 0.04	53 ± 12	2100 ± 100
Z-PLA	1.24 ± 0.06	65 ± 6	4107 ± 211

**Table 3:** Extrusion temperature for 3D filaments containing different TiO_2_ concentrations.

	Filler Concentration, wt.%			
	0	1	3	5
ABS/TiO_2_	260	260	260	280

**Table 4:** Mechanical properties of the irradiated 3D printed composite samples.

Dose, Gy	Specimen	Maximum Load, N	Tensile Stress at Maximum Load, UTS, MPa	Strain at Maximum Load, mm/mm	Tensile Extension at Maximum Load, mm
0	ABS 0% TiO_2_	250.77	25.1	0.0102	0.127
	ABS 1% TiO_2_	254.78	25.5	0.0212	0.531
	ABS 3% TiO_2_	284.43	28.4	0.0211	0.527
	ABS 5% TiO_2_	240.64	24.1	0.0187	0.234
2	ABS 0% TiO_2_	258.05	25.8	0.0197	0.492
	ABS 1% TiO_2_	258.82	25.9	0.0197	0.494
	ABS 3% TiO_2_	249.45	24.9	0.0207	0.517
	ABS 5% TiO_2_	239.63	23.9	0.0189	0.475
30	ABS 0% TiO_2_	255.34	25.5	0.0192	0.480
	ABS 1% TiO_2_	254.42	25.4	0.0210	0.525
	ABS 3% TiO_2_	264.43	26.4	0.0200	0.501
	ABS 5% TiO_2_	252.66	25.3	0.0183	0.458
70	ABS 0% TiO_2_	272.33	27.2	0.0198	0.495
	ABS 1% TiO_2_	251.97	25.2	0.0206	0.514
	ABS 3% TiO_2_	246.35	24.6	0.0191	0.479
	ABS 5% TiO_2_	253.56	25.4	0.0195	0.487

## Data Availability

The data in this work are available upon request from the corresponding author.
